# Distribution and Chemical Nature of Al_2_O_3_ Residues Following the Sandblasting Process: An In Vitro Study Using SEM, EDS, and Cytotoxicity Analysis

**DOI:** 10.1155/bmri/8875067

**Published:** 2026-08-05

**Authors:** Antonio Scarano, Sergio Alexandre Gehrke, Giovanni Falisi, Sergio Rexhep Tari, Tonino Traini, Filiberto Mastrangelo

**Affiliations:** ^1^ Department of Innovative Technology in Medicine and Dentistry, University of Chieti-Pescara, Chieti, Italy, unich.it; ^2^ Zirconia Implant Research Group (Z.I.R.G), International Academy of Ceramic Implantology, Silver Spring, Maryland, USA, iaoci.com; ^3^ Department of Biotechnology, Universidad Católica de Murcia, Murcia, Spain; ^4^ Department of Life, Health and Environmental Sciences, University of L′Aquila, L′Aquila, Italy, univaq.it; ^5^ Department of Clinical and Experimental Medicine, University of Foggia, Foggia, Italy, unifg.it

**Keywords:** aluminum oxide, blasting procedure, contamination, osseointegration, surface cleanliness

## Abstract

**Background:**

Osseointegration is a fundamental requirement for the long‐term success of dental implants. Several implant systems have demonstrated excellent clinical outcomes over time, confirming their high reliability in the treatment of edentulism, even in patients with chronic disease. The success of these devices is closely linked to the characteristics of the implant surface, which directly influence the biological response of the surrounding bone.

**Objectives:**

The primary objective of this study was to investigate the presence of foreign material on the surface of dental implants following aluminum oxide sandblasting and subsequent surface treatment procedures. This analysis was conducted using scanning electron microscopy (SEM) and energy‐dispersive x‐ray spectroscopy (EDS) to characterize the surface morphology and elemental composition. The secondary objective was to evaluate the cytotoxic potential of the implant surface through in vitro assays, in accordance with ISO 10993‐5:2009, to assess its biocompatibility and suitability for clinical application.

**Materials and Methods:**

Twenty threaded screw‐shaped sandblasted Grade 5 titanium dental implants Dental Implant Advance 4.2 × 11.5 mm (Kinetical Implant, Buenos Aires, Argentina) for SEM combined with EDS, in order to identify the presence of possible organic contaminants or residues of the surface treatment process. Another 20 dental implant samples of the same type (4.2 × 11.5 mm) were used for the cytotoxicity test.

**Result:**

SEM analysis revealed a surface morphology consistent with sandblasting treatment, characterized by uniform roughness. However, particles with irregular shapes were observed in certain areas. Aluminum oxide particles were observed adhering to the surface of the implant. The cell viability percentages for each group were as follows: vehicle, 100.0%; negative control (HDPE), 96.0%; positive control (latex), 0.24%; and test samples, 96.9%. Based on the results obtained, the test sample did not show any relevant cytotoxic effects, maintaining a cell viability above 70% and OD values comparable to the negative controls. Therefore, it can be considered noncytotoxic in accordance with ISO 10993‐5.

**Conclusion:**

In conclusion, these findings indicate that the presence of residual alumina particles, resulting from the sandblasting process, does not exert a negative impact on cellular viability. Consequently, such residues can be considered not cytotoxic under the tested conditions, supporting the biocompatibility of the implant surface.

## 1. Introduction

Osseointegration is a fundamental requirement for the long‐term success of dental implants. Several implant systems have demonstrated excellent clinical outcomes over time, confirming their reliability in the treatment of edentulous patients. [1]. The success of these devices is closely linked to the characteristics of the implant surface, which directly influence the biological response of the surrounding bone. In recent years, increasing attention has been devoted to optimizing implant surfaces to improve the interaction between the implant and bone tissue. To this end, numerous surface treatment methods have been developed with the aim of enhancing the predictability of osseointegration and reducing healing times. Among the most common techniques are sandblasting, acid etching, anodization, and the application of bioactive coatings. These approaches represent a key area of innovation in modern implantology, contributing to improved bone–implant contact and promoting faster and more stable healing. Dental implant surfaces are susceptible to a diverse array of organic, inorganic, biological, and procedural contaminants, all of which can impact implant performance and clinical outcomes.

The cleanliness of titanium (Ti) dental implant surfaces is crucial for successful osseointegration and long‐term implant stability. Several studies have identified various components on Ti dental implant surfaces, including organic materials and elements such as carbon, oxygen (O), nitrogen, sodium, calcium, and phosphorus [2, 3]. These components can originate from the manufacturing process, handling, and packaging. For example, sulfur, phosphor, and silicate particles have been observed on abutment surfaces due to laboratory processes [4]. Residual aluminum oxide (Al_2_O_3_) particles from sandblasting processes have been observed. Implants treated with this method showed high levels of osseointegration [5]. The success of Ti dental implants is closely linked to the biocompatibility of their surface oxide layer; in fact, some authors have enhanced the surface oxide layer to improve osseointegration [6, 7]. However, surface contamination, particularly with inorganic substances such as fluorine and aluminum (Al), has been hypothesized to negatively affect osseointegration. Clean implant surfaces are essential for optimal osseointegration, as contaminants can interfere with protein adsorption, cell adhesion, and subsequent bone formation. Impurities such as organic residues and foreign metal particles, often resulting from manufacturing or handling, can negatively impact the biological response and integration process. Cytotoxicity testing is a critical step in evaluating the biocompatibility and safety of dental implant materials. Pure Ti and its alloys (such as Ti‐6Al‐4V) are widely used for dental implants due to their high biocompatibility. Studies show that commercially pure Ti exhibits higher cell viability compared with alloys containing Al and vanadium, which can reduce cell viability due to ion release. However, both materials generally maintain cell viability above 90% at clinically relevant concentrations, and the formation of TiO_2_ over time further decreases cytotoxicity [8].

The primary objective of this study was to investigate the presence of foreign residues on the surface of dental implants following Al_2_O_3_ sandblasting and subsequent surface treatment procedures. This analysis was conducted using scanning electron microscopy (SEM) and energy‐dispersive x‐ray spectroscopy (EDS) to characterize the surface morphology and elemental composition.

The secondary objective was to evaluate the cytotoxic potential of the implant surface through in vitro assays, in accordance with ISO 10993‐5:2009, to assess its biocompatibility and suitability for clinical application.

## 2. Materials and Methods

A total of 40 threaded screw‐shaped sandblasted Grade 5 Ti dental implants (Dental Implant Advance; Kinetical Implant, Buenos Aires, Argentina), produced with high precision equipment, were used in this study. The implants were sandblasted with 200/300 *μ*m Al_2_O_3_ particles at a pressure of 5 atm for 1 min; then, 20 implants underwent the ASTM F86‐68 decontamination process in an ultrasound bath in the following manner:

1. In distilled water for 15 min;
2. In 10% phosphoric acid (H3PO4) for 15 min;
3. In distilled water for 15 min;
4. In 70% nitric acid (HNO3) for 15 min;
5. In distilled water for 15 min.

Twenty samples were used for SEM imaging and elemental analysis. The analysis was conducted at the Biomaterials Research Center of the University of Chieti‐Pescara, Italy. The primary objective was to examine and analyze an implant sample using standardized procedures. This standard defines the requirements for qualitative and semiquantitative surface analysis using SEM combined with EDS, in order to identify the presence of possible organic contaminants or residues of the surface treatment process. All analyses were conducted under controlled conditions, including a laminar flow hood, at standard room temperature.

### 2.1. SEM

**Figure 1:**
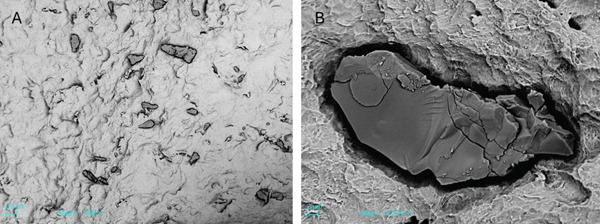
(A) The SEM micrograph shows a particle in close contact with the implant surface. (B) At higher magnification, the morphological adaptation of the particle within a surface depression surrounding the particle.

**Figure 2:**
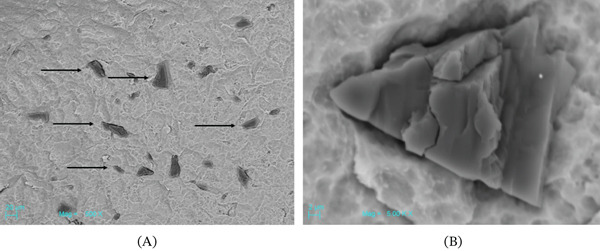
(A) All particles (arrows) contain aluminum in the form of aluminum oxide. No organic material was detected. (B) The morphology of the particles is more clearly discernible at higher magnification.

**Figure 3:**
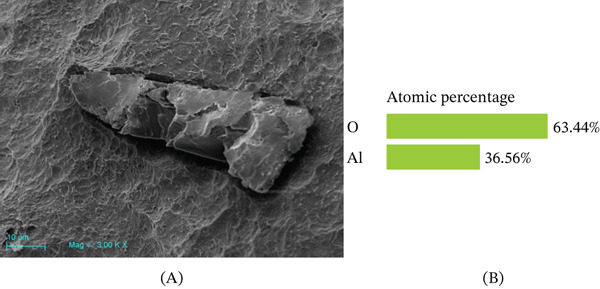
(A) The highlighted particle was analyzed by EDX and revealed a high percentage of oxygen and aluminum, consistent with the presence of aluminum oxide. (B) Quantitative elemental analysis.

The samples were mounted on a conductive stub to improve electrical conductivity. Scans were performed on selected areas of the implant surface, from the apical portion to the coronal region. Images of the implant were acquired at various magnifications (18x, 500x, 3000x, and 5000x), both on the implant body and along the thread profile. SEM images were also captured for specific points of interest (Spot_1, Spot_2, Spot_3, and Spot_4) (Figures 1, 2, and 3).

### 2.2. EDS

EDS was employed for qualitative surface characterization. The EDS investigations were performed on selected surface areas of 20 implants (overview scans) as well as on specific points of interest (Spot_1, Spot_2, Spot_3, and Spot_4).

• A standardless analysis method was used for EDS measurements.
• The report also includes differential EDS analyses, in which x‐ray signals from the bulk material were mathematically subtracted from the total surface signal to estimate the approximate elemental composition of surface particles or thin films.
• Atomic percentages below 1% are provided for orientation purposes only and should not be considered exact values.
• It is important to note that the applicability of the standard is limited for elements with an atomic number (*Z*) lower than 11 (e.g., O), and quantitative data regarding O concentration should be interpreted as approximate.

All analyses were conducted under controlled conditions, including a laminar flow hood, at standard room temperature.

### 2.3. Cytotoxicity Test

The study was designed to evaluate the potential cytotoxic effect of the dental implant (Dental Implant Advance 4.2 × 11.5 mm). A total of 20 implants were used, and each dental implant had a total surface area of 2.2072 cm^2^.

A cytotoxicity test by elution was performed in accordance with the standard ISO 10993‐5:2009.

An extract of the test item was prepared by incubating 20 implant samples in 7.4 mL of supplemented culture medium at 37°C ± 1°C for 72 ± 2 h under dynamic conditions (orbital shaker). The surface area‐to‐volume ratio was 6 cm^2^/mL. The extract was used as is, without filtration, dilution, pH adjustment, or centrifugation. A subconfluent culture of murine fibroblasts BALB/3T3 in the exponential growth phase was used. Cells were seeded in 24‐well plates at a density of 1 × 10^5^ cells/mL and incubated at 37°C ± 1°C in a humidified atmosphere with 5% ± 1% CO_2_ for 24 h to allow monolayer formation. After 24 h, the culture medium was removed and replaced with 0.6 mL of the test extract. Negative controls (HDPE), positive controls (latex), and vehicle controls (supplemented medium) were included. All groups were incubated for an additional 24 h under the same conditions.

Qualitative evaluation: Cell morphology and potential cytotoxic effects were assessed by inverted light microscopy, following the ISO 10993‐5:2009 [9] grading scale (0–4). Cytotoxicity was classified using a 5‐point scale: 0 = *noncytotoxic*, 1 = *slightly cytotoxic*, 2 = *moderately cytotoxic*, 3 = *markedly cytotoxic*, and 4 = *severely cytotoxic*. Optical density (OD) values and the coefficient of variation (CV%) were calculated to describe the central tendency and variability of the data, as the results are expressed as indices on a categorical classification scale (Table 1).

**Table 1:** ISO 10993‐5:2009 cytotoxicity grading scale.

Grade	Reactivity	Description of cellular response
0	None	Discrete intracytoplasmic granules; no cell lysis; no reduction in cell growth. Not more than 20% of the cells are round, loosely attached, and without granules
1	Slight	Intracytoplasmic granules or slight morphological changes; occasional lysed cells; slight growth inhibition. Not more than 50% of the cells are round and devoid of granules
2	Mild	No extensive cell lysis; not more than 50% growth inhibition. Not more than 70% of the cell layer shows rounding or lysis
3	Moderate	Cell layers are not completely destroyed, but more than 50% growth inhibition is observed
4	Severe	Nearly complete or complete destruction of the cell layers

*Note:* Cytotoxicity was classified using a 5‐point scale: 0 = *noncytotoxic*, 1 = *slightly cytotoxic*, 2 = *moderately cytotoxic*, 3 = *markedly cytotoxic*, and 4 = *severely cytotoxic*.

Quantitative evaluation: The neutral red uptake (NRU) assay was used to assess cell viability, based on the ability of viable cells to incorporate and bind the vital dye neutral red. After incubation, cells were exposed to the dye for 3 h, lysed with a desorb solution, and absorbance was measured at 540 nm using a microplate reader. Cell viability was expressed as a percentage relative to the vehicle control. The biological reactivity (cell degeneration and malformations) was evaluated after 24 h of incubation with a scale ranging from 0 to 4, according to ISO 10993‐5, as shown in Table 1. Cytotoxicity was evaluated using an in vitro cell viability assay in accordance with ISO 10993‐5:2009 guidelines. To ensure the reliability and interpretability of the results, the following controls were included in the experimental design:

Blank control: This condition contained neither cells nor reagents and was used to measure the optical or metabolic background of the system.

Vehicle control: Comprised only of cells cultured in the extraction medium or solvent used for sample preparation. This control was employed to assess any potential cytotoxic effects attributable to the vehicle itself.

Negative control: Included polystyrene, a material well‐documented for its biocompatibility, to confirm the absence of cytotoxic effects under standard conditions.

Positive control: Contained polyvinyl chloride (PVC), a material known for its cytotoxic properties, to verify the sensitivity and responsiveness of the assay system.

All samples and controls were tested in triplicate, and OD values were measured spectrophotometrically. The CV% was calculated to assess the reproducibility of the measurements.

### 2.4. Statistical Analysis

The analysis of the cytotoxicity assay was limited to descriptive statistics. Results are reported as mean values ± standard deviation obtained from experimental replicates. No inferential statistical tests were performed, as the assessment of cytotoxicity was conducted in accordance with ISO 10993‐5:2009, which defines biocompatibility based on predefined cell viability thresholds rather than on statistical significance.

## 3. Results

### 3.1. SEM

SEM analysis revealed a surface morphology consistent with sandblasting treatment, characterized by uniform roughness. However, isolated particles with irregular shapes were observed in certain areas. Al_2_O_3_ particles were observed on the surface of the implant. Particles of Al_2_O_3_ have not been detected simply adhering to the implant surface in the absence of such morphological alteration.

### 3.2. EDS

**Figure 4:**
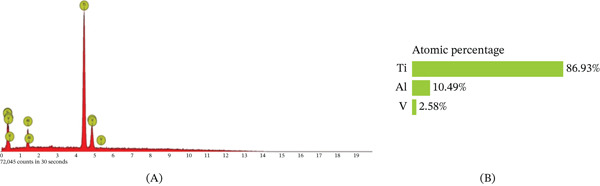
(A) The EDX analysis of the entire surface revealed the presence of titanium, vanadium, and aluminum. (B) The elemental composition was quantified in terms of atomic percentages, highlighting the distribution of these elements across the analyzed area.

The test results revealed that the implant samples exhibited numerous aluminum oxide particles uniformly distributed across the surface, with sizes ranging from 10 to 70 *μ*m. Elemental analysis of these particles detected only signals corresponding to Al and O, confirming their inorganic nature (Figure 4).

No organic or carbon‐based particles were identified within the magnification range used (500x–5000x), indicating a clean surface in terms of organic contamination, within the limits of the applied analytical resolution. The analysis should be considered qualitative and not intended as a quantitative assessment of all samples.

EDS analysis confirmed the presence of Al in correspondence with these particles, suggesting the persistence of aluminum oxide residues not completely removed by the subsequent surface treatment procedure. Additionally, elements consistent with the composition of Ti and traces of O were detected, in line with the presence of the native surface oxide layer.

### 3.3. Cytotoxicity

#### 3.3.1. Qualitative Evaluation

Following 24 h of incubation with the test item extract, cell cultures were examined under an inverted light microscope to assess morphological changes indicative of cytotoxicity. In the wells treated with the extract of the test item (Dental Implant Advance 4.2 × 11.5 mm), cells exhibited discrete intracytoplasmic granules. No evidence of cell lysis or inhibition of cell proliferation was observed. According to the ISO 10993‐5:2009 grading scale, the observed reactivity was classified as Grade 0, indicating no cytotoxic response.

#### 3.3.2. Quantitative Evaluation

After the qualitative assessment, cells were incubated for 3 h with neutral red–containing medium to evaluate viability. After dye uptake, cells were lysed using a desorb solution, and the OD of the resulting solution was measured at 540 nm using a spectrophotometer.

The test item extract induced a 3% reduction in cell viability compared with the vehicle control. Based on the criteria defined in ISO 10993‐5:2009 [9], which considers a viability reduction greater than 30% as indicative of cytotoxicity, the test samples were classified as noncytotoxic, as shown in Table 1. The cell viability percentages for each group were as follows: vehicle, 100.0%; negative control (HDPE), 96.0%; positive control (latex), 0.24%; and test samples: 96.9%.

### 3.4. Statistical Analysis

Descriptive statistical analysis of the cytotoxicity assay showed that the test samples exhibited a mean cell viability of 96.9% compared with the vehicle control. The negative control demonstrated comparable viability values, whereas the positive control induced a pronounced reduction in cell viability. Based on ISO 10993‐5:2009 guidelines, which define cytotoxicity as a reduction in cell viability greater than 30%, the tested implant surface was classified as noncytotoxic.

## 4. Discussion

The present study demonstrated the presence of aluminum oxide particles uniformly distributed across the surface of sandblasted Ti dental implants, with particle sizes ranging from 10 to 70 *μ*m, as observed by SEM and confirmed by EDS analysis. These findings are consistent with previous reports describing residual alumina particles following sandblasting surface treatments. SEM imaging revealed aluminum oxide particles associated with localized surface irregularities. However, SEM provides surface morphological information only and does not allow definitive conclusions regarding particle embedding, cavity formation, or the mechanisms by which these particles are retained on the implant surface. SEM/EDS analysis was conducted as a qualitative surface characterization on 20 implants. The observations were intended to document the presence and morphological appearance of residual particles, rather than provide a quantitative assessment of implant‐to‐implant variability or production process consistency. Cytotoxicity assays performed in compliance with ISO 10993‐5:2009 revealed no evidence of cellular toxicity, confirming the biocompatibility of the tested material [9]. Elemental analysis of these particles only detected Al and O, confirming their inorganic nature.

No organic, that is, carbon‐based, particles were detected within the magnification limits used (500x–5000x), suggesting the absence of visible carbonaceous contamination at these scales. However, the analysis should be considered qualitative and not intended as a quantitative assessment of all samples.

The role of implant surface contamination in implant failure is not yet fully understood. However, it is hypothesized that contaminants may be released from the implant surface, triggering an inflammatory response that could compromise the healing process. Al_2_O_3_ (alumina) particles are commonly found on implant surfaces, primarily as bioinert remnants from sandblasting processes, and as such should not be rated as contaminants. These particles can be present in high quantities and across a wide size range. It is reasonable to assume that the presence of surface contaminants may influence surface free energy and hydrophilicity, both of which are crucial for cell attachment and spreading. Contaminated surfaces are associated with decreased bone‐to‐implant contact and impaired tissue integration, especially on rougher surfaces. Cleaner or higher surface energy implants show improved fibroblast activity and integration, whereas contaminated surfaces are associated with increased risk of peri‐implantitis and compromised healing [10]. It has been reported in the literature that Al ions can inhibit the normal differentiation of bone marrow stromal cells, impair bone matrix deposition and mineralization [11], and induce calcium loss in bone cultures [12]. However, aluminum oxide (Al_2_O_3_), or alumina, is widely recognized as a bioinert and practically insoluble material, which makes it generally safe for biomedical applications. The scientific literature indicates that aluminum oxide (Al_2_O_3_) can influence osseointegration through its effects on implant surface properties, cell behavior, and bone–implant interactions. In a canine model, Al was shown to inhibit the formation of calcium phosphate crystals and the growth of poorly crystallized hydroxyapatite. Some studies report that residual Al_2_O_3_ particles do not significantly affect osseointegration or bone–implant contact, suggesting that the effect may depend on particle amount, distribution, or other surface factors [5, 13]. Recent studies have shown that residual aluminum oxide (Al_2_O_3_) on the surface of Ti dental implants can actually enhance surface wettability and bone tissue growth and support osseointegration. Notably, when present at approximately 8% by weight, alumina appears to promote a more favorable biological response, potentially improving the integration of the implant with surrounding bone tissue [14].

Al is a lightweight, ductile metal widely used in industry due to its workability and corrosion resistance. Its oxide, aluminum oxide (Al_2_O_3_), is instead an extremely hard ceramic material, chemically and thermally stable, nonconductive, and biocompatible, properties that make it ideal for applications in the medical, electronic, and engineering fields. Alumina is an advanced ceramic material widely used in medicine, particularly for joint prostheses, dental implants, and biomedical devices, due to its excellent biocompatibility, high hardness, and outstanding wear resistance. Under neutral biological conditions, alumina is insoluble and does not release significant Al ions. Al ions may significantly inhibit hydroxyapatite growth by altering the morphology of calcospherites. Initially, they reduce their average diameter and subsequently transform them into less structured, calcified plaques [15, 16]. Al is the most abundant metal in the Earth′s crust and is always found combined with other elements such as O, silicon, and fluorine. Its compounds are widely used in various sectors, including water treatment, abrasives, refractory coatings, and consumer products such as antacids, cosmetics, antiperspirants, and medical devices. Alumina‐blasted and alumina‐blasted/acid‐etched surfaces increase surface roughness, which correlates with improved osseointegration and biomechanical fixation compared with machined surfaces [17]. Alumina is biocompatible and supports osteoblast adhesion and proliferation, but its bioinert nature may limit direct bone growth unless surface modifications are applied [18]. Alumina ceramics have been studied in various forms for a wide range of applications. In orthopedic surgery, they have been used for many years due to their excellent biocompatibility, minimal fibrous capsule formation, and low friction and wear rates. Although trace amounts of Al ions were detected in the surrounding medium, no adverse effects on cellular activity were observed; in fact, an increase in osteoblastic growth was observed, particularly during the early stages of bone healing [18]. However, surface chemistry and inadequate cleanliness of dental implant surfaces are strongly associated with the development of peri‐implantitis, underscoring the critical importance of evaluating and maintaining surface purity to ensure long‐term implant success [19]. The presence of additional elements in alloys and surface modifications can influence cytotoxicity. Alloys with fewer additional elements tend to have lower cytotoxic effects, and proper surface treatments (such as sandblasting and passivation) can enhance biocompatibility [20]. Cytotoxicity is commonly assessed using MTT assays, LDH assays, and other cell viability tests on fibroblasts or osteoblastic cell lines. International standards such as ISO 10993‐5 and ISO 7405 guide these evaluations, ensuring consistency and reliability in results [21]. In conclusion, the implants tested in this study can be considered free of organic contaminants. Moreover, the presence of residual alumina on Ti implant surfaces may enhance bone tissue growth and stimulate osteoblast activity by modifying surface energy and wettability. However, it is important to note that some studies have reported no significant impact on bone‐to‐implant contact. Based on the results obtained and interpreted in accordance with ISO 10993‐5:2009, the tested implants must be classified as noncytotoxic. The results of the present study demonstrated that the tested implant surfaces did not induce any cytotoxic effects, as confirmed by in vitro assays conducted in accordance with ISO 10993‐5:2009. These findings indicate that the presence of residual alumina particles, resulting from the sandblasting process, does not exert a negative impact on cellular viability. Consequently, such residues can be considered not cytotoxic under the tested conditions, supporting the biocompatibility of the implant surface.

## Funding

Open access publishing facilitated by Universita degli Studi Gabriele d’Annunzio Chieti Pescara, as part of the Wiley ‐ CRUI‐CARE agreement.

## Conflicts of Interest

The authors declare no conflicts of interest.

## Data Availability

The data that support the findings of this study are available on request from the corresponding author.
